# Distinct metabolic profiles in *Drosophila* sperm and somatic tissues revealed by two-photon NAD(P)H and FAD autofluorescence lifetime imaging

**DOI:** 10.1038/s41598-019-56067-w

**Published:** 2019-12-20

**Authors:** Cornelia Wetzker, Klaus Reinhardt

**Affiliations:** Technische Universität Dresden, Faculty Biology, Applied Zoology, D-01069 Dresden, Germany

**Keywords:** Cell biology, Zoology, Optics and photonics

## Abstract

Metabolic profiles vary across all levels of biological diversity, from cells to taxa. Two-photon fluorescence lifetime imaging microscopy (FLIM) facilitates metabolic characterisation of biological specimens by assaying the intrinsic autofluorescence of the ubiquitous coenzymes NAD(P)H and FAD. The potential of this method for characterising the diversity of organismal metabolism remains largely untapped. Using FLIM in *Drosophila melanogaster*, we show tissue-specificity in fluorescence lifetime that reflects variation in redox patterns. In particular, sperm cells exhibited elevated glycolysis relative to other tissues. We also show that sperm metabolism is phenotypically plastic: compared to male-stored sperm, sperm stored in the female’s storage organ showed a substantial reduction in the protein-bound FAD lifetime fraction but no change in the NAD(P)H profile. This study represents the first *ex vivo* investigation of sperm metabolism using FLIM.

## Introduction

Sperm cells are highly motile in many taxa, propelling the male genome to the oocyte. On their way through the female reproductive tract, the energy to fuel their motility must be provided, at least in part, internally, through metabolism. However, few studies have clarified the role of sperm metabolism in sperm quality and male fertility. Because female insects are able to store sperm for extended periods of time, in some species up to decades^1^, insects prove particularly eligible for the study of sperm physiology in the diverse natural environments being male and female reproductive tissues.

The molecular unit of energy, adenosine triphosphate (ATP), is produced mainly from upstream metabolites by glycolysis in the cytoplasm and oxidative phosphorylation (OxPHOS) at the inner mitochondrial membrane. Cells tightly control the balance of OxPHOS vs. glycolysis, since OxPHOS is highly efficient but can also produce damaging reactive oxygen species (ROS), whereas glycolysis produces no ROS but also little ATP. The reduction/oxidation (redox) cofactors nicotinamide adenine dinucleotide (NAD^+^/NADH) and flavin adenine dinucleotide (FAD/FADH_2_) are key players in cellular metabolism. Glycolysis consumes glucose and reduces NAD^+^ to NADH, independently of oxygen. The resulting pyruvate is fed into the citric acid cycle, in which NAD^+^ and FAD are further reduced to increase mitochondrial levels of the electron carriers NADH and FADH_2_. These coenzymes are the driving force for OxPHOS, and hence their redox status reflects energy metabolism.

Cellular NADH and FAD can be assayed optically, by taking advantage of their autofluorescent properties. This label-free approach allows rapid *in vivo* or *ex vivo* imaging without the need for transgenic animals. Briefly, oxidised FAD and reduced NAD(P)H (representing NADH and its spectrally indistinguishable phosphorylated variant NADPH^2–4^) are autofluorescent, but their redox counterparts NAD(P)^+^ and FADH_2_ are not in a similar way^5^.

A particularly sensitive optical character of NADH and FAD autofluorescence are their lifetimes, i.e., the duration of the molecules’ transition from the excited state to the ground state. Importantly, the autofluorescence lifetime is responsive to the fluorophore’s chemical environment^6,7^, including interacting proteins. For example, unbound NADH, being free of interacting proteins and thus internally folded, has two described fluorescence lifetimes of 300 to 800 picoseconds (ps) and approximately 1000 ps^7–10^. Protein-bound NAD(P)H, chemically in an extended conformation, has longer lifetimes, between 1000 and 3000 ps depending on the protein^7–9,11–14^. For FAD, the unbound form has a fluorescence lifetime ranging from 1700 to 3200 ps, falling to 40 to 650 ps upon binding to proteins^15–17^. NAD(P)H fluorescence lifetimes can be used to infer the metabolic pathways operating in a cell: unbound NAD(P)H has been assigned mainly to glycolytic metabolism, whilst protein binding may indicate interaction with enzymes including those of the mitochondrial electron transport chain and, therefore, OxPHOS^18^. This allows the characterisation of the metabolic state of biological samples based on the relative fractions of unbound and protein-bound NAD(P)H.

Fluorescence lifetime data can be further graphically visualised and analysed in phasor plots^18–21^. To generate these plots, raw lifetime decay data of each pixel in an image are transformed fit-free and mapped to a position in a two-dimensional plot. Clustering of pixels with similar metabolic behaviour and their relative positions allows for a more complex interpretation of FLIM data.

The cellular metabolic state can be further evaluated independently of fluorescence lifetime parameters by incorporation of NAD(P)H and FAD concentrations. The optical redox ratio (ORR) is defined as the ratio of fluorescence intensities of the two coenzymes ([FAD]/[NAD(P)H])^22,23^, or, in a normalised form [FAD]/([NAD(P)H] + [FAD])^24^. The ORR increases with increasing rates of OxPHOS: NADH levels decrease due to the oxidation of NADH to NAD^+^, while FAD levels increase due to the oxidation of FADH_2_ to FAD. Recently, a lifetime-based redox measure based on NAD(P)H and FAD, termed the FLIM redox ratio (FLIRR), has been introduced^25^. This measure is defined as the ratio of the fraction of NAD(P)H that is protein-bound (i.e., with a long lifetime) to the fraction of FAD that is protein-bound (i.e., with a short lifetime). Similar to the ORR, FLIRR increases with higher metabolic activity^25^: the fraction of protein-bound NAD(P)H, associated with electron transport chain enzymes, increases, while non-fluorescent FADH_2_ is converted to unbound fluorescent FAD, thereby reducing the fraction of protein-bound FAD.

For more than a decade, FLIM has been applied for metabolic monitoring in stem cell (e.g.^13,17,18,23,26,27^) and cancer research (e.g.^16,28–31^). Both types of cells are characterised by a more glycolytic status compared to differentiated and non-tumorigenic cells, respectively, as revealed by higher ratios of unbound-to-bound NAD(P)H and hence shorter mean NAD(P)H lifetimes.

Here, we advocate for the use of FLIM to study the metabolism of sperm cells, for which the balance between glycolysis and OxPHOS is critically important. Sperm must metabolise sufficient energy for motility whilst limiting ROS damage to the cell membrane and the paternal genome that may otherwise lead to infertility in humans and other animals^32–34^. Moreover, females of many species and across the animal kingdom can store fertile sperm for extended periods, in some species for several years^35^. Yet it is not clear how damage to sperm is being prevented over such long periods of time. Studies in insects suggest that a switch in sperm metabolism after being transferred from the male to the female may be involved^36–39^. If so, very different metabolic fingerprints should be expected in sperm taken from the male vs. the female environments. Here, we first establish the feasibility of FLIM to characterise metabolism in a variety of tissues of the fruit fly *Drosophila melanogaster* (*D*. *melanogaster*). We then use this method to evaluate how sperm metabolism is modified after transfer to the female’s storage organ. We identify striking shifts in the FLIRR based mainly on FAD but not NAD(P)H changes.

## Results

### NAD(P)H autofluorescence lifetimes and phasor plots identify tissue-specific metabolic signatures in *D*. *melanogaster* organs

We characterised the metabolism of tissues of *D*. *melanogaster*, including i) salivary gland (SGCs) and fat body cells (FBCs) of third instar larvae, ii) enterocytes of the posterior midgut of adult females, and iii) sperm in male and female storage organs. Application of NAD(P)H and FAD FLIM using two-photon laser excitation has proved particularly advantageous for investigations of living tissues. Compared to one-photon excitation, two-photon excitation applies light of longer wavelength and allows for increased imaging quality through reduced tissue damage and interference, as well as increased imaging depth.

**Figure 1:**
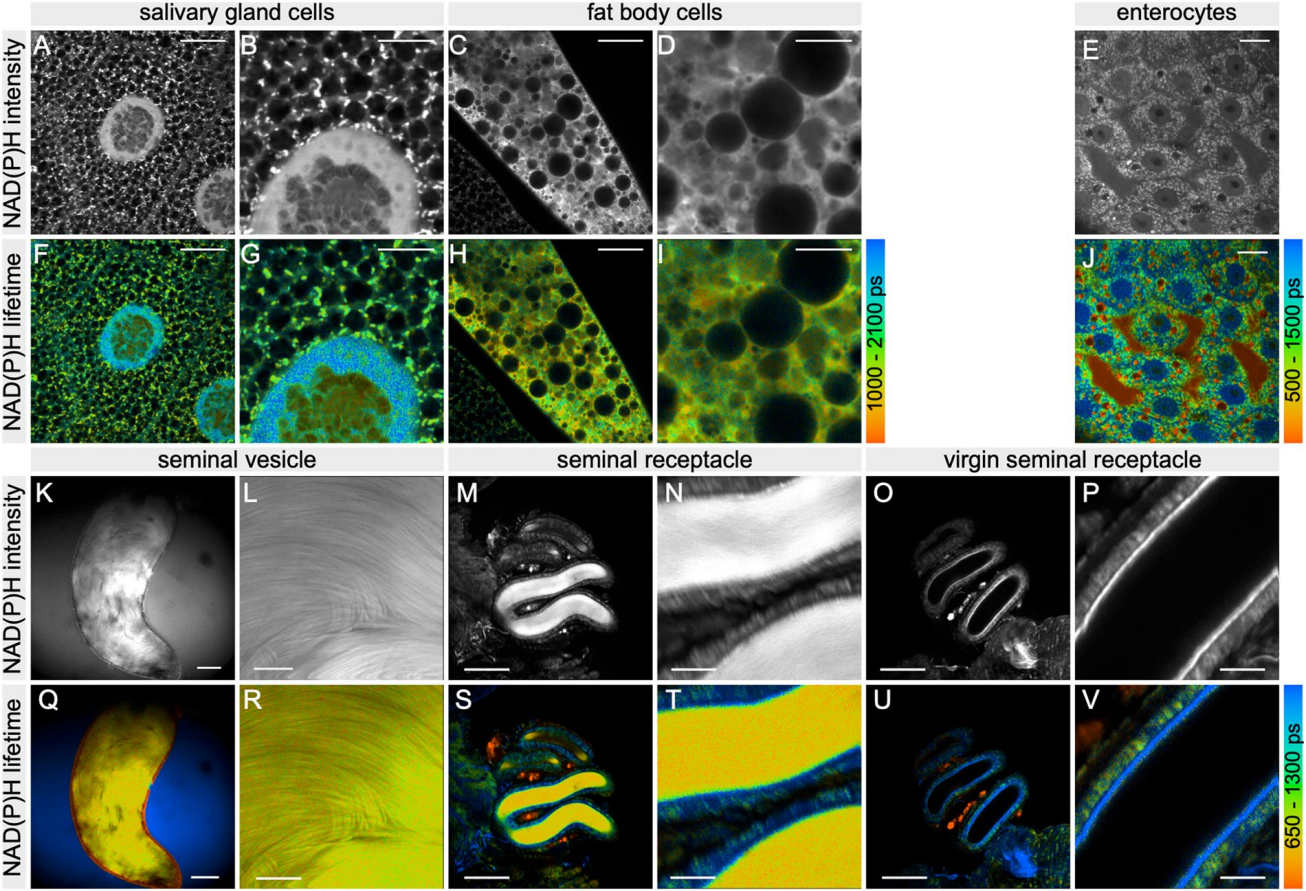
NAD(P)H lifetime imaging of *D*. *melanogaster* tissues. NAD(P)H intensity (rows 1 and 3) and pseudo-colour mean NAD(P)H lifetime (rows 2 and 4) images are shown at low and higher magnification for salivary gland cells (**A**,**B**,**F**,**G**) and fat body cells (**C**,**D**,**H**,**I**) of third instar larvae, adult midgut enterocytes (**E**,**J**) and sperm in the male’s seminal vesicles (**K**,**L**,**Q**,**R**) and in the female’s seminal receptacle of a mated (**M**,**N**,**S**,**T**) and a virgin control (**O**,**P**,**U**,**V**) fly. The virgin seminal receptacle specimen is devoid of background signal in the tubular lumen. Scale bars are 50 *μ*m (**K**,**M**,**O**,**Q**,**S**,**U**), 10 *μ*m (**A**,**C**,**E**,**F**,**H**,**J**) and 5 *μ*m (**B**,**D**,**G**,**I**).

We initially investigated the metabolism of larval tissues and generated two-dimensional false-color mean NAD(P)H lifetime images (Fig. 1F–I). SGCs of third instar larvae showed distinct NAD(P)H lifetime signatures in a variety of distinguishable subcellular structures, including mitochondria, the nucleoplasm, and chromatin. NAD(P)H FLIM thus proved feasible for the metabolic investigation of living *Drosophila* larval tissues. We further studied enterocytes of the posterior midgut subregion P2^40^ of adult females (Fig. 1J). FLIM proved applicable to adult insect tissues, and metabolic differences based on NAD(P)H lifetimes revealed subcellular structures in enterocytes.

We then investigated sperm cells stored in the bag-shaped, paired seminal vesicles as part of the male’s reproductive tract prior to mating. After mating, female fruit flies store sperm for several days to several weeks in two highly specialised organs: the long, coiled seminal receptacle and the paired spermathecae. Sperm displayed a similar NAD(P)H lifetime pattern when stored in the male’s seminal vesicle as when stored in the female’s seminal receptacle (Fig. 1Q–T). In both sexes, NAD(P)H FLIM assigned the sperm mass a homogenous metabolic pattern clearly distinct from that of the surrounding epithelia. In the male’s seminal vesicle, the epithelium showed a dramatically shorter mean lifetime due to the presence of melanin (Fig. 1Q). In the twisted tubes of the female’s seminal receptacle, sperm are surrounded by an epithelial cell layer of intermediate lifetime lined with a structurally distinct layer^41^ of longer lifetime (Fig. 1S,T). The strong melanin signal in the spermathecae of *D*. *melanogaster* females prevented sperm metabolic imaging altogether. In both males and females, the needle-shaped sperm heads containing the nuclei could not be morphologically distinguished from the sperm tails, likely because of the extreme DNA compaction. The sperm-free seminal receptacle of unmated females illustrated the lack of background signal from out-of-focus planes and further confirmed the identification of sperm in mated females (Fig. 1U,V).

**Figure 2:**
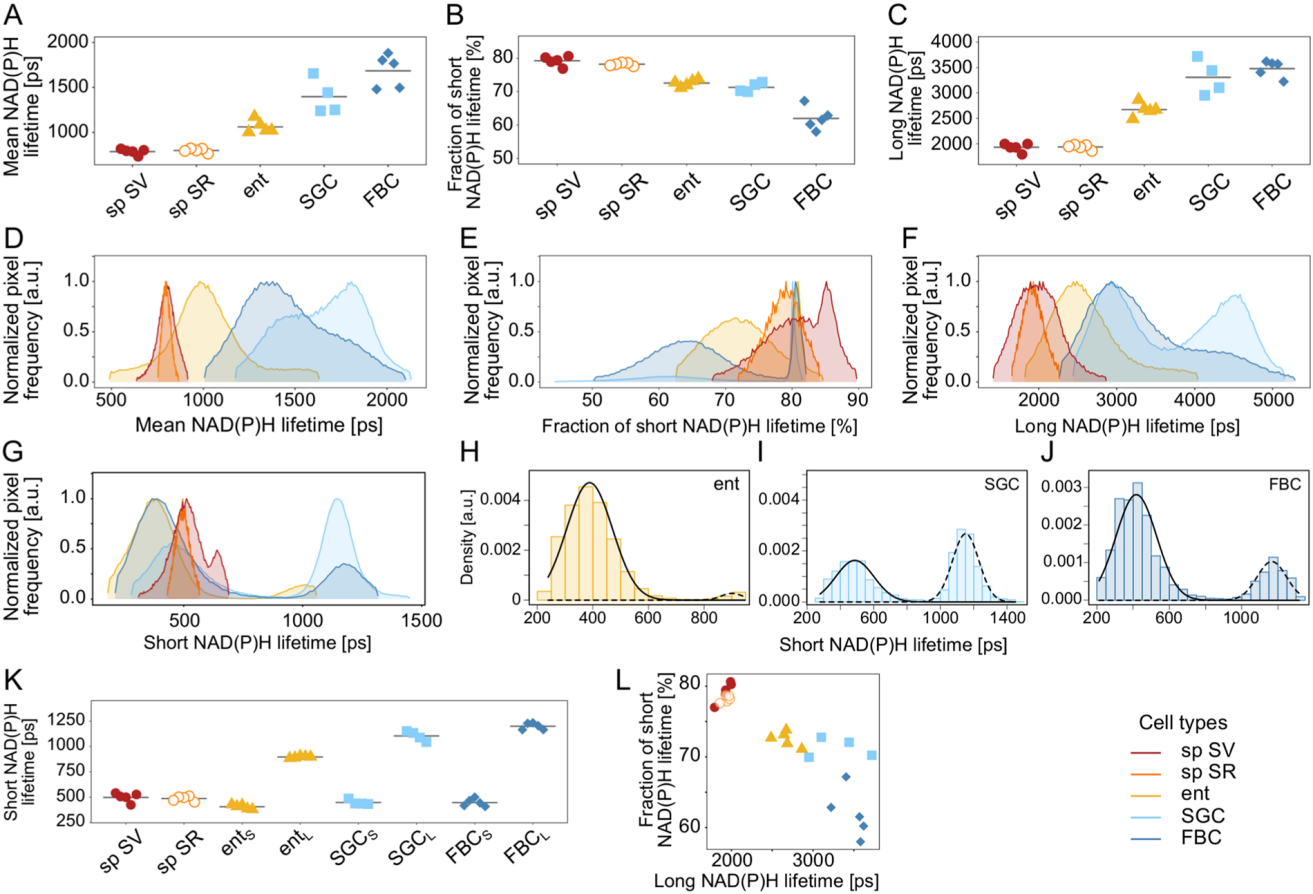
Graphical visualisation of NAD(P)H lifetime parameters of *D*. *melanogaster* tissues. Mean values of NAD(P)H mean (**A**) and long (**C**) lifetimes and the fractions of short (NAD(P)H lifetime (**B**) of image regions of interest as single data points for all samples and tissue means as well as the respective distributions (**D**–**F**) for a single sample of each investigated *D*. *melanogaster* tissue are shown. The short NAD(P)H lifetime distributions of enterocytes, salivary gland (SGCs) and fat body cells (FBCs) displayed two peaks (**G**). Distribution histograms and two separated populations (solid and dashed lines) of short NAD(P)H lifetimes of individual samples are displayed for enterocytes, SGCs, and FBCs (**H**–**J**). The means of each population are shown individually along with single short NAD(P)H lifetime means from sperm samples (**K**). Tissues clustered in a scatter plot of the short vs. long NAD(P)H lifetime fractions (**L**). Sp SV, sperm in seminal vesicles; sp SR, sperm in seminal receptacle; ent, enterocytes.

We further quantified the metabolic signatures of all tissues by comparing their NAD(P)H lifetime parameters and distributions (Fig. 2). In salivary glands and enterocytes, nuclei were identified and excluded from lifetime data extraction to avoid the interference with non-metabolic NAD(P)H signals originating from gene expression in the nucleus (Suppl. Fig. 1). For the analysis of sperm metabolism, regions representing sperm were subselected in the images and then in the phasor plots prior to FLIM data extraction (Suppl. Fig. 2). SGCs showed a mean lifetime (±SD) of 1395.97 ± 195.73 ps and FBCs of 1684.92 ± 185.76 ps, enterocytes of 1060.45 ± 70.66 ps, sperm of 785.41 ± 33.25 ps in the male’s and 798.82 ± 23.25 ps in the female’s storage organs (Fig. 2A) (Sample sizes N = 4 males for SGCs, N = 5 for all other tissues). The free NAD(P)H contributed 71.26 ± 1.38% to the mean fluorescence lifetime in SGCs and 61.96 ± 3.42% in FBCs, 72.69 ± 1.06% in enterocytes, 79.23 ± 1.44% in sperm in the male, and 78.19 ± 0.48% in sperm in the female (Fig. 2B). The variation within tissues was smaller than across tissues (Fig. 2B).

Long lifetime means differed strongly across tissues, being 3300 ± 350 ps in SGCs, 3480 ± 170 ps in FBCs, 2670 ± 130 ps in enterocytes, 1930 ± 80 ps in male-stored sperm, and 1940 ± 50 ps in female-stored sperm (Fig. 2C). The frequency distributions of lifetime means in the somatic cells showed broad peaks, particularly in the larval cells. The presence of two peaks in the SGCs indicated metabolic differences in their cytoplasmic regions (Fig. 2F). Tissue differences in all three NAD(P)H lifetime parameters were clearly observed in the frequency distributions (Fig. 2D–F). Overall, the low mean NAD(P)H lifetime and the high contribution of unbound NAD(P)H indicate that sperm cells are highly glycolytic compared to the investigated somatic tissues. SGCs and FBCs were in a metabolic state most associated with OxPHOS, while enterocytes showed an intermediate metabolic condition.

The frequency distributions of short NAD(P)H lifetimes showed two distinct peaks in the somatic cells, but only a single prominent peak in sperm (Fig. 2G). We consequently subdivided the short lifetimes of SGCs, FBCs, and enterocytes into two distinct populations per cell type (Fig. 2H–J). The means of the short lifetime peaks in sperm were 497.99 ± 45.32 ps in males and 486.88 ± 25.22 ps in females (Fig. 2K). Short lifetime populations of the non-sperm cell types had means (±SD) of 405.93 ± 21.07 ps and 895.72 ± 8.61 ps for enterocytes, 448.85 ± 25.58 ps and 1104.56 ± 48.34 ps for SGCs, and 447.69 ± 37.22 ps and 1199.97 ± 32.54 ps for FBCs. There was a clear negative relationship across cell types (though not necessarily within cell types) between the long NAD(P)H lifetime and the fraction of the short lifetime. Data points clustered cell type-specifically (Fig. 2L).

**Figure 3:**
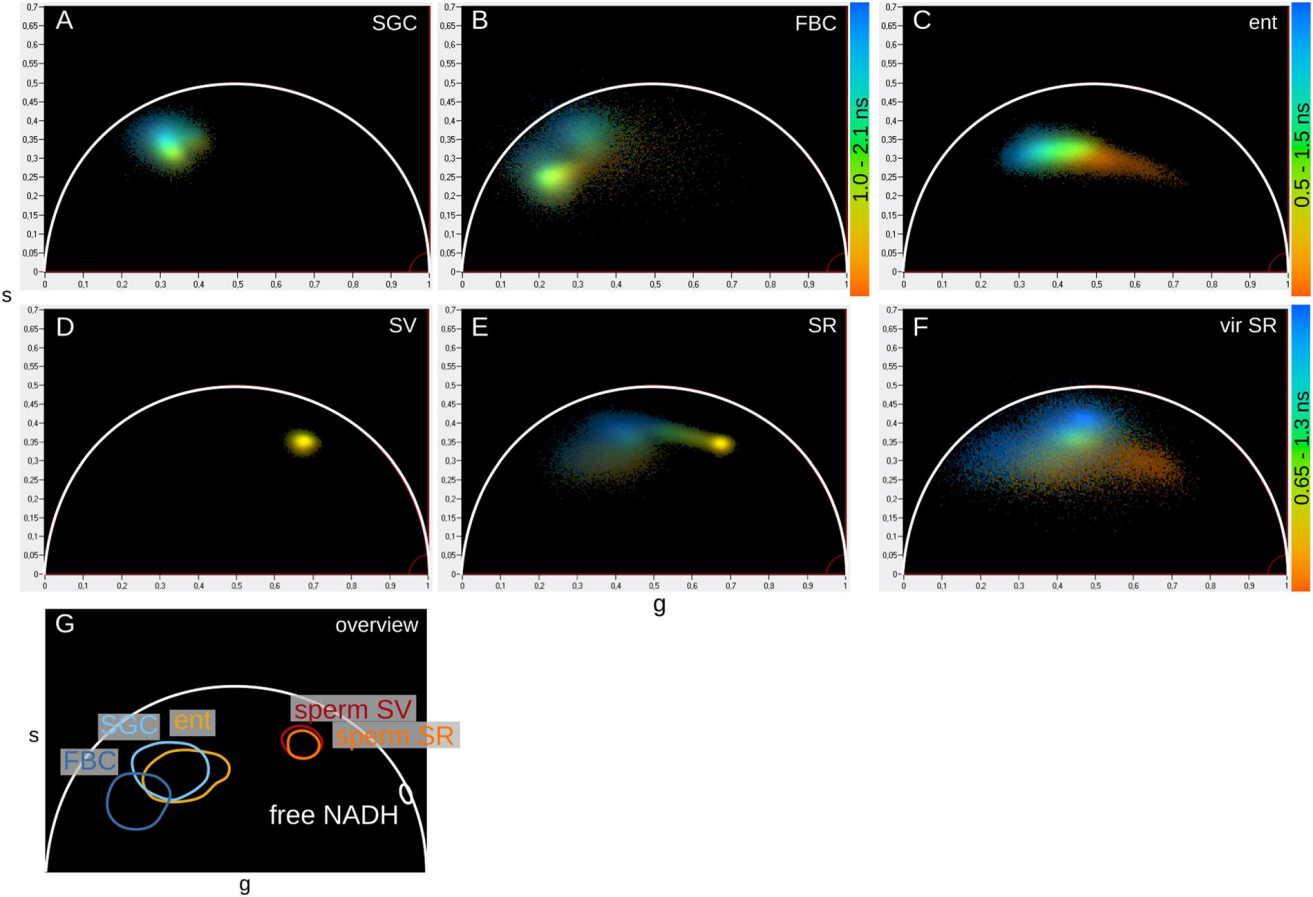
NAD(P)H phasor plots of *D*. *melanogaster* tissues. FLIM phasor plots display individual tissue-specific patterns for SGCs (**A**) and FBCs (**B**) of a third instar larva, enterocytes of an adult female (**C**), sperm in a seminal vesicle (**D**), part of the seminal vesicle containing sperm (**E**), and a virgin seminal receptacle control (**F**). Each pixel of an image is localized to a position below the universal semicircle (white) according to its fluorescence lifetime decay. A schematic overview of the cluster positions of the individual plots relative to unbound NADH in solution emphasises cell type-specific differences, with sperm in the seminal vesicle and the seminal receptacle in nearly identical positions farthest to the right and larval SGCs and FBCs in the farthest left position (**G**). SV, seminal vesicle; SR, seminal receptacle; vir SR, virgin seminal receptacle; ent, enterocytes; SGC salivary gland cells; FBC, fat body cells.

Each tissue exhibited a global cell type-specific NAD(P)H lifetime phasor plot signature that complements the two-exponential decay analysis because it makes no assumption about the number of lifetime components. The clusters of the somatic tissues all localised left of the center of the plot, indicating a higher fraction of protein-bound NAD(P)H (Fig. 3A–C). Signatures of sperm stored in males and females both clustered in the same location, right of the center of the plot closest to the unbound NADH, i.e. sole in solution, cluster reflecting the highest short lifetime fractions (Fig. 3D,E). The seminal receptacle of sperm-devoid, unmated females clearly lacked this cluster (Fig. 3F). These data show that sperm pixel clusters were most closely associated with unbound NAD(P)H, characteristic of a highly glycolytic state^18^ (Fig. 3G). SGC and FBC clusters associated most strongly with the bound NAD(P)H phasor positions reported in other studies, indicative of a strong oxidative state. Enterocytes were in an intermediate state.

### NAD(P)H mean lifetime distributions are tissue-specific metabolic fingerprints

**Figure 4:**
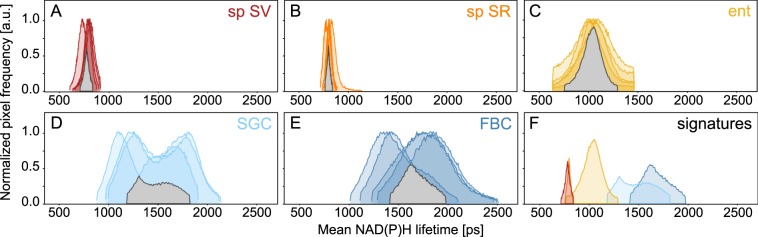
Tissues-specific mean NAD(P)H lifetime signatures. Mean NAD(P)H lifetime histograms of biological replicates for each investigated tissue as overlap patterns (grey regions) show limited variability within tissues (**A**–**E**). Comparison of these tissue-specific areas of overlap emphasizes the different cell type-specific metabolic patterns (**F**). Sp SV, sperm in seminal vesicles; sp SR, sperm in seminal receptacle; ent, enterocytes; SGC, salivary gland cells; FBC, fat body cells.

Each investigated tissue was characterised by a specific mean NAD(P)H lifetime distribution largely conserved across biological replicates (Fig. 4). Sperm and enterocytes showed a single sharp mean NAD(P)H lifetime peak, which was highly similar but not identical between sperm in male and in female storage organs (Fig. 4A–C). SGCs and FBCs exhibited larger variability between biological replicates and a broader mean lifetime peak due to this higher cross-sample heterogeneity, possibly because more subcellular structures are visible in these than sperm cells (Fig. 4D,E). Again, as for the long lifetime component, the biological replicates showed a reproducible core range representing clearly distinct, cell type-specific NAD(P)H lifetime signatures (Fig. 4F). Each overlap area allocates a specific NAD(P)H lifetime signature to the various cell types and tissues.

### Sperm cells stored in males and females differ in protein-bound FAD fractions and the FLIM redox ratio

**Figure 5:**
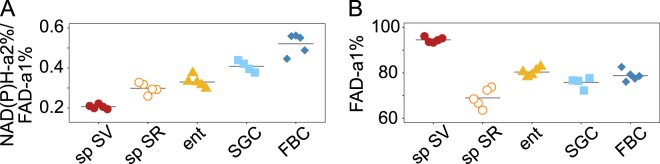
FLIM redox ratio (FLIRR) of *D*. *melanogaster* tissues. Mean FLIRR values calculated as NAD(P)H-a2%/FAD-a1% of ROIs of images of all investigated *D*. *melanogaster* tissues and group means display a different FLIRR for each investigated cell type and also for sperm stored in males (sp SV) and females (sp SR) (**A**). The latter difference is highlighted in the FAD-a1% plot of region of interest means grouped by tissue, with large differences between sperm stored in different biological environments in *D*. *melanogaster*, while all somatic tissues show intermediate values (**B**). ent, enterocytes; SGC, salivary gland cells; FBC, fat body cells.

Mean FLIRR values (±SD), the lifetime-based measure of the redox ratio, clearly differed between sperm in males (0.21 ± 0.01) and females (0.30 ± 0.03), with no overlap between these groups (Fig. 5A). The FLIRR of enterocytes was 0.33 ± 0.03, of SGCs 0.41 ± 0.03, and of FBCs 0.52 ± 0.05. While NAD(P)H lifetime metrics were highly similar in sperm located in both sexes, differences in the FLIRR imply different contributions of protein-bound FAD. In fact, sperm stored in males vs. in females showed the largest mean difference of protein-bound FAD fractions across the investigated insect tissues, with values of 93.59 ± 1.15% in seminal vesicles and 66.69 ± 4.14% in seminal receptacles (Fig. 5B). Somatic tissues showed intermediate contributions of protein-bound FAD, with values of 79.06 ± 1.80% in enterocytes, 75.39 ± 1.41% in SGCs, and 77.47 ± 2.42% in FBCs.

## Discussion

In the present work, we demonstrate the feasibility of two-photon FLIM of the autofluorescent cellular biomarkers NAD(P)H and FAD to assess the metabolic states of different *D*. *melanogaster* tissues. Our study represents the first investigation of insect sperm metabolism in intact, living organs; previous studies investigated sperm after its release from the storage organs^36,37,39^. We found that i) sperm were highly glycolytic, in contrast to somatic tissues (Figs. 1–4); ii) the metabolic fingerprints resulted in distinct, likely machine-readable profiles that offer themselves for clinical testing; and iii) the distinct differences in sperm metabolism found in the male and female storage organ of *D*. *melanogaster* were, unlike in other animals, caused by differences in FAD, not NAD(P)H.

Sperm of *D*. *melanogaster* were highly glycolytic, compared to somatic tissues (Figs. 1–4). Somatic tissues in our study clearly showed distinct patterns including NAD(P)H lifetimes, phasor distributions, and mean lifetime fingerprints indicative of more oxidative metabolism (Figs. 1–4). For example, we found high OxPHOS activity in larval SGCs, an observation that is in agreement with their high oxygen consumption observed prior to pupariation^42^. We also note that the three parameters - not independent but based on different analyses - largely agreed with one another. The diversity of patterns of long NAD(P)H lifetimes (Fig. 2F) suggests different protein interactions as described in other studies^14^, and consequently different biochemical networks across various tissues.

While the mode of ATP production in sperm differs across species with regard to the balance between glycolysis and OxPHOS, the unusually high glycolytic activity recorded here contrasts with the general view of mitochondria representing the powerhouses of sperm. In human^43^ and rodent sperm, glycolysis predominates, as in *Drosophila* in this study, while OxPHOS and glycolysis coexist balanced in cattle sperm^44^. In principle, NAD(P)H autofluorescence FLIM is applicable to the sperm of these species, although there are some differences between our approach and other current methods that require consideration. First, in contrast to mammalian sperm, *D*. *melanogaster* sperm reside in the female reproductive tract for several weeks rather than a few days. In the future, it would be of interest to measure the metabolic profile of sperm during long-term storage in female insects. In addition, sperm metabolism has usually been examined in sperm collected from the male, which has limited power to predict the performance in the female^36,45^. Furthermore, on a potentially related note, according to established protocols^46^ sperm is exposed to ambient levels of oxygen after collection - levels that are likely never encountered in the female reproductive tract. Importantly, both caveats are circumvented with our method. Future research on potential clinical applications of our method should test how metabolism differs in sperm exposed to ambient oxygen levels compared to physiological oxygen levels in the female. After such a calibration, it will be useful to quantify aberrations of sperm metabolism, e.g. NAD(P)H and FAD lifetime parameters, in clinical cases of infertility.

Finally, there is a marked difference in morphology between mammalian and *D*. *melanogaster* sperm. Unlike mammalian sperm, insect sperm commonly lack a mitochondria-containing midpiece. Instead, they have a mitochondrial derivative that extends through the entire length of the sperm tail^47^. Its detailed structure, function, and capacity for oxidative metabolism are still unresolved. Protein or peptide degradation, indicated, e.g., by a high abundance of leucine aminopeptidases in *D*. *melanogaster* mitochondrial derivatives^48,49^, are possible but likely not the sole mechanisms for energetic supply of sperm in males and females. Because these enzymes only partially clarify a predominance towards glycolytic or oxidative metabolism, two-photon FLIM presents itself as a promising method to analyse the structural composition and mechanistic functions of mitochondrial derivatives in insect sperm.

We identified core areas of mean NAD(P)H lifetime signatures that did not overlap between tissues and so may serve as tissues-specific markers (Fig. 4). The identification of such cores, as well as the estimation of their variation across individuals has important technological implications. We thus propose that, given the rapidly advancing development of neural networks for pattern recognition, tissue-specific lifetime and phasor distributions have the potential to serve as machine-readable metabolic FLIM signatures. Such automated pattern recognition and analysis may advance a broad range of applications from basic research to clinical diagnosis and therapy^50^. The non-invasive diagnosis of cancerous tissue based on FLIM, such as melanoma tissue in humans^51^, has been described earlier.

Previous studies examining the metabolism of sperm stored in males and females agreed that there was a relative increase in glycolysis in female-stored sperm compared to ejaculated, or male-collected, sperm. This was shown in crickets (*Gryllus bimaculatus*)^36^, and bedbugs (*Cimex lectularius*) using the unbound-to-bound NAD(P)H ratio^37^, as well as in honeybees (*Apis mellifera*), in which glycolysis was predominant in sperm in the queen’s oxygen-depleted spermatheca while ejaculated sperm also underwent mitochondrial respiration^39^. Another study, examining bedbug sperm metabolism based on mean NAD(P)H lifetimes, did not find differences between male- and female-stored sperm, but did reveal larger variation in females^38^. Interestingly, we also discovered no differences between male- and female-stored sperm if the analysis was restricted to NAD(P)H lifetimes. We did, however, find a difference in the protein-binding behaviour of FAD: the fraction of protein-bound FAD was dramatically lower in female-stored compared to male-stored sperm (Fig. 5). Because the fraction of protein-bound FAD (FAD-a_1_%) is the denominator of the redox indicator FLIRR, a lower FAD-a_1_% causes a higher FLIRR and indicates higher metabolic activity^25^ in sperm in female compared to male storage. It is clear from these results that the FAD-related biology of *D*. *melanogaster* sperm is altered considerably upon transfer to the female’s seminal receptacle. The relative importance of metabolic effects including fatty acid beta-oxidation and other cellular FAD-involving processes, such as electron transport, and synthesis of cofactors and coenzymes, as well as DNA repair and nucleotide biosynthesis^52^, remains to be tested. For example, protein-bound FAD decreases during tumorigenesis in epithelial cells^16^ and is also affected lineage-specifically upon mesenchymal stem cell differentiation^17^. Increased beta-oxidation of fatty acids has been proposed to decrease protein-bound FAD levels^23^, and may be the cause of low protein-bound FAD levels in larval FBCs in our study.

In this work, we demonstrate that two-photon NAD(P)H and FAD FLIM can be used to characterise metabolic states of intact insect tissues, including, for the first time, sperm in their natural physiological environment. While the diversity of tissues that can be investigated will likely necessitate sample-specific adaptations of data analysis pipelines to incorporate morphological and mechanistic characteristics, the core parameters identified here will facilitate automated data processing. Finally, our work extends the applicability of metabolic two-photon autofluorescence FLIM beyond differentiation-, tumorigenesis-, and disease-associated states to investigate the role of environmental conditions on the metabolism of cells, tissues, and whole organisms.

## Methods

### Fly maintenance and tissue dissections

Isogenic *D*. *melanogaster* CantonS flies were provided by Marko Brankatschk (MPI-CBG Dresden, Germany) and outbred *D*. *melanogaster* of the wild-type white Dahomey (wDah) strain by Adam Dobson (University College, London, UK). Midguts were derived from wDah females and all other tissues from the CantonS strain. Flies were maintained at 25 °C in an incubator with a 12 h light:dark cycle. For CantonS flies, the diet was a standard medium containing 90 g/l corn meal, 40 g/l yeast, 100 g/l sucrose, 12 g/l agar, 40 ml/l nipagin (10% in ethanol) and 3 ml/l propionic acid in water. The diet for wDah contained 100 g/l yeast, 50 g/l sucrose, 15 g/l agar, 30 ml/l nipagin (10% in ethanol) and 3 ml/l propionic acid in water. Adult male and female flies were kept together in vials to allow repeated matings. Control virgin females were isolated within four hours after eclosion. Prior to dissections, flies were placed in 70% ethanol for approximately 30 seconds and then washed briefly with PBS. The reproductive tracts, including seminal vesicles in males and seminal receptacles in females, were dissected in PBS with fine forceps from adult flies aged five to seven days using a binocular microscope. Seminal vesicles were carefully disentangled from testicular structures by removing the connective fibers between structures in order to allow imaging in a focal plane. Midguts of 7-day-old females were dissected and imaged in CyGel (BioStatus, Shepshed, UK) to reduce peristaltic movements. Salivary glands, comprising the glands themselves and the associated fat body tissue, were dissected from third instar larvae. All samples were placed in 40 *μ*l of solution on an object slide, covered with a coverslip resting on tiny clay feet, and sealed with nail polish. Imaging of all organs was performed within a maximum of ten minutes after dissections. Tissues and individual cells were located and identified using transmitted light microscopy before metabolic imaging.

### FLIM measurements

Reduced NAD(P)H absorbs ultraviolet light of approximately 340 nm with one-photon excitation and 740 nm with two-photon excitation and emits light in the range of 460 nm^3,53–56^. FAD absorbs light of 900 nm with two-photon excitation and emits light in the range of 500 to 550 nm^5,12,56,57^. Intracellular NAD(P)H and FAD-based metabolic data was generated using time-correlated single photon counting (TCPSC) FLIM^58^. The microscope setup includes an upright AxioExaminer.Z1 (Carl Zeiss, Jena, Germany) with an xy-motorized stage, a Chameleon Ultra II two-photon titanium:sapphire laser (tunable range 690–1080 nm, 80 MHz repetition rate, 140 fs pulse width, Coherent, USA) and two hybrid GaAsP photon detectors (HPM-100-40, Becker&Hickl GmbH, Berlin, Germany). Prior to imaging, the organs were localised by transmission light illumination. For NAD(P)H and FAD lifetime imaging, fluorescence was two-photon excited with light of 740 nm followed by 900 nm, respectively. Light was focused on the analysed optical planes using a water immersion objective (LD C-Apochromat 40x/1.1 W, 421867-9970-000, Carl Zeiss, Jena, Germany) for all measurements. 10 *μ*M NADH dipotassium salt (N4505, Sigma-Aldrich, Germany) in PBS was used for the measurement of unbound NADH.

Tissue damage and the saturation of the FLIM detection system were prevented by the limitation of the total photon count rates to a maximum of 8 × 10^5^ per second by laser power limitation to approximately 2.6 to 11.5 mW for 740 nm excitation and 10.4 to 39.0 mW for 900 nm excitation in the sample position by the internal acusto-optical modulator (AOM, Becker&Hickl GmbH, Berlin, Germany) measured with a powermeter (PM100A, ThorLabs, Dachau, Germany). Emitted light was spectrally split for specific detection with a beam splitter of 505 nm combined with synchronous emission of band pass-restricted spectral ranges of 450/30 nm and 525/39 nm (beam splitter and filters from AHF analysentechnik AG, Tübingen, Germany) for NAD(P)H and FAD, respectively. Images of different zoom factors and 512 × 512 pixel format were acquired using SPCM software version 9.80 (Becker&Hickl GmbH, Berlin, Germany) with a pixel dwell time of approximately 5 *μ*s and 100 frames yielding a total scan time of approximately 180 s. 6 *μ*m fluorescent beads were imaged for size estimates.

### Fluorescence lifetime data analysis

The lifetimes of intracellular NAD(P)H and FAD were fitted two-exponentially with non-fixed parameters short lifetime (*τ*_1_) and long lifetime (*τ*_2_), a fixed scatter of “0” and fixed shift values for each image using SPCImage software version 6.5 (Becker&Hickl GmbH, Berlin, Germany). Mean NAD(P)H and FAD lifetimes *τ*_*m*_ were calculated by addition of the products of *τ*_1_ or *τ*_2_ with their respective fractions, a_1_% (unbound for NAD(P)H and protein-bound for FAD) and a_2_% (protein-bound for NAD(P)H and unbound for FAD) (*τ*_*m*_ = *τ*_1_ × a_1_% + *τ*_2_ × a_2_%) by the software.

Pixel selection according to localisation in the 2D images and phasor plots is shown using representative examples in the Supplementary information (Suppl. Figs. S1 and S2). In detail, for all cell types, regions of interest (ROIs) were selected manually in images of mean NAD(P)H lifetime with a defined colour range in order to exclude surrounding tissues. In images of enterocytes, SGCs, and FBCs, nuclei, if distinguishable, were also excluded from the ROIs. ROIs of images of NAD(P)H lifetime values were applied to corresponding images of FAD lifetime values. The means of the following lifetime parameters were exported: for NAD(P)H, the mean lifetime, the short and long lifetimes, and the fraction of the short lifetime; and, for FAD, the fraction of the short lifetime. The distribution of binned data from each sample was exported from SPCI for distribution histograms and for the separation of two populations for the short NAD(P)H lifetimes in enterocytes, SGCs, and FBCs. All statistics were performed, and all plots excluding the phasor plots were generated, in R (version 3.5.1) using RStudio (version 1.1.456). Labels were adjusted and distribution overlays generated using Inkscape (version 0.92). Identification of lifetime populations from the short lifetime distribution data of the ROIs of individual samples was performed by application of a mixed model (normalmixEM) using the mixtools package in R with the assumption of two clusters.

### Phasor approach of lifetime analysis

The phasor plots represent fit-free graphical views of intensity decays and are calculated as described earlier using the SPCI software^20^. In detail, each point of the phasor plot is calculated by sine (s) and cosine (g) transformation of the fluorescence decay trace of each pixel of the image using the following equations:

1 $${g}_{i,j}(\omega )=\frac{{\int }_{0}^{\infty }\,{I}_{i,j}(t)\,\cos (\omega t)dt}{{\int }_{0}^{\infty }\,{I}_{i,j}(t)dt}$$

2 $${s}_{i,j}(\omega )=\frac{{\int }_{0}^{\infty }\,{I}_{i,j}(t)\,\sin (\omega t)dt}{{\int }_{0}^{\infty }\,{I}_{i,j}(t)dt}$$

The indices i and j are pixels of the image and s_*i*,*j*_ (*ω*) and g_*i*,*j*_ (*ω*) are y and x coordinates, respectively, of the phasor plot. The laser repetition frequency was 80 MHz in our experiments. The fluorescence collected from each pixel of an image was thus transformed to a point in the phasor plot. The phasor localisation integrates the complexity of the decay and the individual lifetimes for each pixel of a FLIM image. Single lifetime species locate on the universal semicircle along which the lifetime decreases with increasing g on the x axis. Multiexponential lifetime species in which several decay behaviours localise vectorially added within the polygons defined by the contributing individual lifetimes species. The FLIM redox ratio (FLIRR) was calculated as the ratio of NAD(P)H-a_2_% and FAD-a_1_% with NAD(P)H-a_2_% as a_2_/(a_1_ + a_2_) of NAD(P)H parameters and FAD-a_1_% as a_1_/(a_1_ + a_2_) of FAD parameters^25^.

## Supplementary information

Supplementary Information

## Data Availability

The FLIM raw and processed data (.sdt and .img files) are stored in the OpARA repository, 10.25532/OPARA-37. All other data generated and analysed during the current study are available from the corresponding author upon request.
