# Supplementary material for: Distinct metabolic profiles in Drosophila sperm and somatic tissues revealed by two-photon NAD(P)H and FAD autofluorescence lifetime imaging
Source: Sci Rep. 2019 Dec 20;9:19534. doi: 10.1038/s41598-019-56067-w (PMC6925207; doi:10.1038/s41598-019-56067-w)
Supplement: Supplementary file 1 — Supplementary Information [file 41598_2019_56067_MOESM1_ESM.pdf]

Supplementary Figures

Distinct metabolic profiles in *Drosophila* sperm and somatic tissues revealed by two-photon NAD(P)H and FAD autofluorescence lifetime imaging

Cornelia Wetzker and Klaus Reinhardt

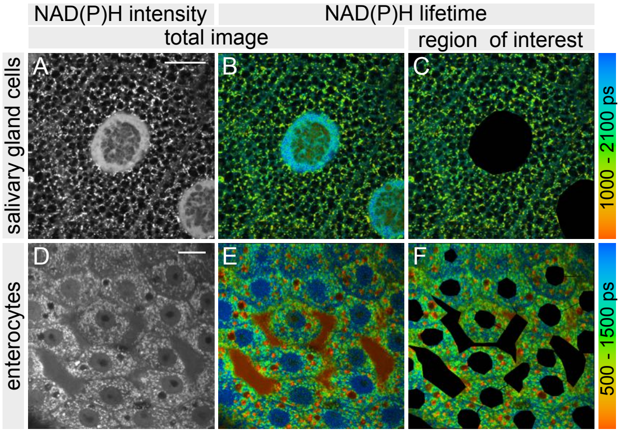

Fig S1. Pixel selection from NAD(P)H lifetime images of *D. melanogaster* salivary gland cells and enterocytes. NAD(P)H intensity images (A, D) and colour-coded lifetime images (B, C, E, F) are displayed for salivary gland cells (top row) and enterocytes (bottom row). Regions containing nuclei and sinoids were excluded from total lifetime images (B, E) to yield pixels used for lifetime data extraction (C, F). Scale bars are 10 μm.

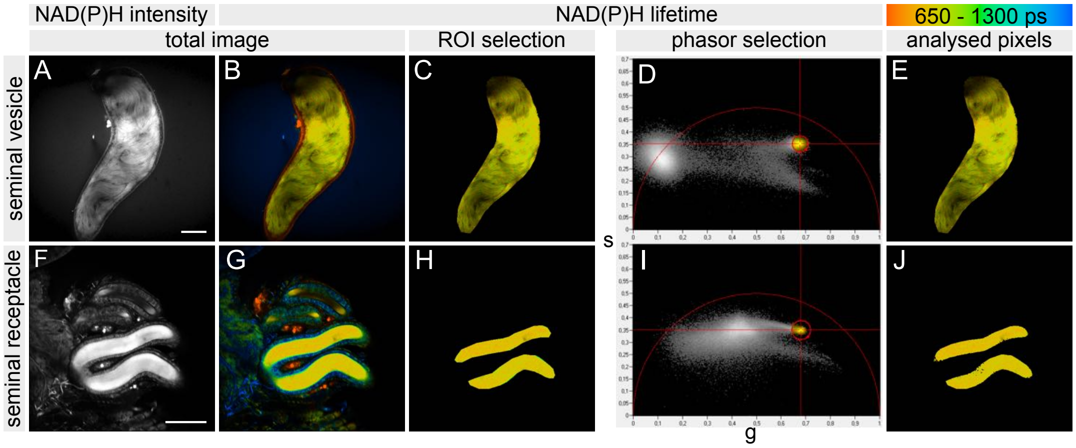

Fig S2. Pixel selection from NAD(P)H lifetime images of sperm storage organs. NAD(P)H intensity images (A, F) and colour-coded lifetime images (B, C, E, G, H, J) and phasor plots (D, I) are displayed for a seminal vesicle (top row) and a seminal receptacle (bottom row). ROIs (C, H) based on total lifetime images (B, G) are combined with cluster selection from phasor plots (D, I) to yield the pixels used for lifetime data extraction (E, J). Scale bars are 50 μm.
